# Hyperthyroidism in gestational trophoblastic disease – a literature review

**DOI:** 10.1186/s13044-021-00092-3

**Published:** 2021-01-14

**Authors:** Jarett Vanz-Brian Pereira, Taylor Lim

**Affiliations:** Faculty of Medicine, University of New South Wales, Wallace Wurth Building - UNSW Sydney, 18 High St, Kensington NSW, Sydney, 2052 Australia

**Keywords:** Gestational trophoblastic disease, Molar pregnancy, Hyperthyroidism, Hydatidiform mole, Pregnancy, Thyroid, HCG, Chorionic gonadotropin

## Abstract

**Objective:**

Gestational trophoblastic disease (GTD) is a group of pregnancy-related disorders that arise from abnormal proliferation of placental trophoblast. Some patients with GTD develop hyperthyroidism, a rare but potentially life-threatening complication requiring early detection and management. Existing literature on hyperthyroidism in GTD is scant. This review aims to analyse the epidemiology, pathophysiology and management of this phenomenon.

**Methods:**

A comprehensive search of MEDLINE, EMBASE and Cochrane Library was performed to obtain articles that explored hyperthyroidism in GTD. A total of 405 articles were screened and 228 articles were considered for full-text review. We selected articles that explored epidemiology, pathophysiology and outcomes/management of hyperthyroidism in GTD.

**Results:**

The pathophysiology of hyperthyroidism in GTD is well-investigated. Placental trophoblastic tissue secretes excessive hCG, which is structurally similar to thyroid stimulating hormone and also has enhanced thyrotropic activity compared to normal hCG. The incidence and prevalence of hyperthyroidism in GTD varies worldwide, with lower rates associated with high uptake of early antenatal screening and early GTD detection. No clear risk factors for hyperthyroidism in GTD were identified. While hyperthyroidism can be definitively managed with surgical evacuation of the uterus, severe complications associated with hyperthyroidism in GTD have been reported, including thyroid storm-induced multi-organ failure, ARDS, and pulmonary hypertension.

**Conclusion:**

Early detection of GTD is critical to prevent development of hyperthyroidism and its associated complications. Hyperthyroidism should be recognised as an important perioperative consideration for women undergoing surgery for GTD, and requires appropriate management. Future studies should explore risk factors for hyperthyroidism in GTD, which may facilitate earlier identification of high-risk women.

**Supplementary Information:**

The online version contains supplementary material available at 10.1186/s13044-021-00092-3.

## Introduction

Gestational trophoblastic disease (GTD) is the umbrella term for a heterogenous group of pregnancy-related disorders arising from abnormal proliferation of placental trophoblast. GTD encompasses the premalignant condition of hydatidiform mole (complete and partial) and gestational trophoblastic neoplasia (GTN). Invasive mole, choriocarcinoma, placental site trophoblastic tumour (PSTT), and epithelioid trophoblastic tumour (ETT) are all classified under GTN [1]. It is challenging to accurately determine the epidemiology of GTD due to its rarity, varying clinical definitions and the shortage of centralised databases [1, 2]. The incidence of benign GTD is estimated to be 1/1000 pregnancies [3], while choriocarcinoma occurs in approximately 1/40,000 pregnancies. As PSTT and ETT only make up 0.2% of GTD cases, epidemiological data is scant [4].

GTD was historically associated with significant morbidity and mortality. Hydatidiform mole was often complicated by severe bleeding prior to the development of early detection and effective uterine evacuation techniques, while invasive mole had a mortality rate of 15% due to bleeding, embolisation of trophoblastic tissue and sepsis. Metastatic choriocarcinoma was also universally fatal [5]. Effective uterine evacuation techniques and the use of chemotherapy today have significantly improved survival, and trophoblastic tumours are now highly-curable. Other complications of GTD include pre-eclampsia, hyperthyroidism, hyperemesis and respiratory distress [6].

Despite being a rare complication of GTD, hyperthyroidism can lead to life-threatening clinical consequences when present, therefore requiring early detection and treatment. However, the early diagnosis of hyperthyroidism can be challenging because of its rarity and the low level of suspicion among clinicians. The diagnosis of hyperthyroidism may also be missed if the hypermetabolic symptoms are simply attributed to the trophoblastic disease [7]. Furthermore, with uterine evacuation being the mainstay of management for hydatidiform mole and GTN, hyperthyroidism represents an important perioperative consideration as thyrotoxicosis can be fatal [8]. In the setting of emergency surgery, cardiovascular manifestations of hyperthyroidism may also be wrongly attributed to hypovolemia and the diagnosis of hyperthyroidism can be missed [9].

To the best of our knowledge, review articles on hyperthyroidism in GTD are scant. Most of the cited evidence regarding hyperthyroidism in GTD are individual case reports. Therefore, a comprehensive overview of the available evidence is timely and necessary. This literature review aims to summarise the evidence regarding the epidemiology, pathophysiology and outcomes of hyperthyroidism in GTD.

## Methods

### Literature search

A comprehensive electronic search of MEDLINE, EMBASE and the Cochrane Library was performed to obtain articles published from database inception to 13th October 2019. The search strategy was formulated using a combination of MeSH terms, controlled vocabulary, and Boolean phrase capabilities, including: “gestational trophoblastic”, “molar pregnancy”, “hydatidiform mole”, “trophoblastic neoplasms”, “hyperthyroidism”, “thyroid storm” and “thyrotoxicosis”. Restrictions were not applied; we searched for all studies that explored hyperthyroidism in GTD. In addition, reference lists of search results were scanned to identify additional publications not retrieved by the electronic search. The complete search strategy for each database is presented in Additional file 1. All references were uploaded into EndNote (v.X7) and duplicate articles were removed.

### Study selection

Articles retrieved from the search were screened for eligibility based on title and abstract using the Rayyan platform [10]. We included articles that explored hyperthyroidism and GTD. The following were excluded: 1) studies that solely looked at either hyperthyroidism or GTD; 2) articles on non-gestational choriocarcinoma and 3) articles on pregnancies with mole and coexistent foetus.

### Search results

**Fig. 1:**
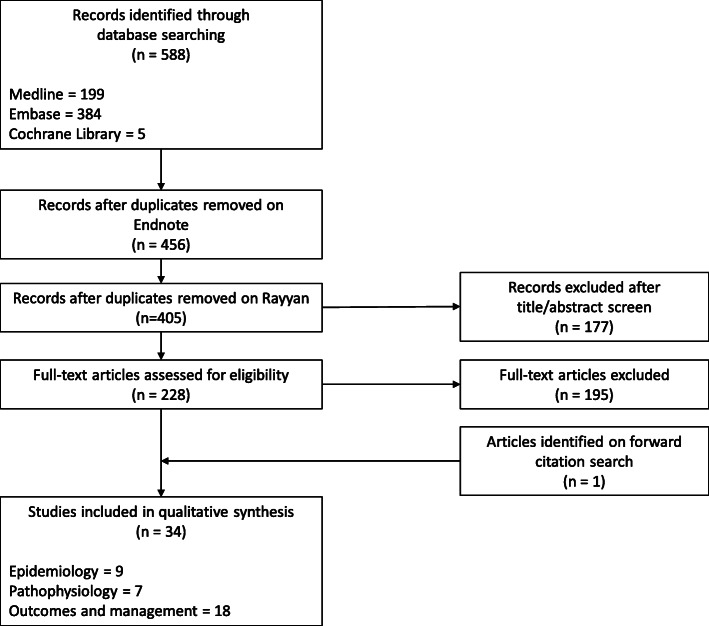
Flow diagram outlining the identification and selection of articles

A total of 456 articles were retrieved by our electronic search, after removal of duplicates on Endnote. An additional 51 duplicates were identified on the Rayyan platform, resulting in 405 unique articles undergoing title and abstract screen. Of these articles, 177 were excluded based on the exclusion criteria. In total, 228 articles were considered for the review. An outline of the search and article selection process is shown in Fig. 1.

Of the 228 articles, most were case reports on the occurrence of hyperthyroidism or thyrotoxicosis in patients with GTD. No relevant systematic review, meta-analysis or randomised trial topic was identified. We further shortlisted articles that fit into either one of the following categories: “epidemiology”, “pathophysiology” and “outcomes/complications/management”. In total, 18 articles on “outcomes/complications/management”, 9 articles on “epidemiology” and seven articles on “pathophysiology” were used for this literature review.

### The epidemiology of hyperthyroidism in GTD

#### Setting

Due to its rarity, few studies have attempted to determine the incidence of hyperthyroidism in GTD. Here we explored the incidence of GTD-induced hyperthyroidism in various settings, and a summary is provided in Table 1.

**Table 1:** Epidemiology of hyperthyroidism in GTD across various settings

Study details			GTD type	Total number of GTD cases reviewed in study	Number of patients with hyperthyroidism	
Authors	Year	Population and setting			Biochemical (% of total GTD cases)	Clinical (% of total GTD cases)
Walkington, et al. [11]	2011	Patients commencing chemotherapy for GTN at Weston Park hospital, Sheffield, United Kingdom, between January 2005 and January 2010.	GTN	196	14 (7.2)	4 (2)
Sun, et al. [12]	2016	Patients with CM and PM from the Trophoblastic disease centre, United States of America, between 1994 and 2013	CM	194	31 (16)	4 (2.1)
			PM	172	8 (4.7)	4 (2.3)
Norman, et al. [13]	1981	Black patients with GTD admitted to the gynaecological wards of King Edward VIII Hospital, South Africa, over a 12-month period	GTN	27	15 (56)	9 (33)
Al Alaf, et al. [14]	2010	Patients with GTD admitted to Maternity Teaching Hospital, Erbil City, Iraq, from 1st October 2008 to 1st April 2009	GTN	3	10 (25) ^a^	
			CM	33		
			PM	4		
Bahasadri, et al. [15]	2011	Patients with CM or PM admitted to Akbarabadi Teaching Hospital, Tehran, Iran, between 21st June 1996 and 3rd July 2006	CM	230	10 (4.3) ^a^	
			PM	34	0 (0) ^a^	

^a^There was no distinction between biochemical and clinical hypothyroidism in the study

*GTD* Gestational trophoblastic disease

*GTN* Gestational trophoblastic neoplasia

*CM* Complete hydatidiform mole

*PM* Partial hydatidiform mole

A study based in a dedicated GTD centre in Sheffield (United Kingdom) reviewed 196 patients who were treated with chemotherapy. Of these 196 patients, 14 (7.2%) had biochemical hyperthyroidism (based on TSH, FT3 and FT4), and four (2%) had clinical hyperthyroidism [11]. Given that the management of GTN in the UK is highly centralised and patients usually present early in gestation, the rates of hyperthyroidism in this study may be lower than other less-resourced healthcare settings.

A US study analysed one of the largest gestational trophoblastic cancer registries in the United States [12]. The authors identified 194 women with histopathologically confirmed complete mole (CM) and 172 with partial mole (PM). More women with CM developed biochemical hyperthyroidism compared to PM (16% vs 4.7%; *p* < 0.001). However, only 4/194 (2.1%) and 4/172 (2.3%) of women from the CM and PM had clinical hyperthyroidism, respectively. Overall, the incidence of hyperthyroidism in this cohort was approximately similar to the previously-described Sheffield cohort.

A 1981 South African study reported higher rates of hyperthyroidism in their cohort of GTD patients [13], with 15/27 (56%) developing biochemical hyperthyroidism. Clinical hyperthyroidism was seen in 9/27 (33%). The higher incidence of hyperthyroidism in this study compared to the previous two is most likely explained by later detection of GTD in women, compared to the other studies. Being a significantly older study, it is plausible that early detection techniques were either less developed or had lower uptake/implementation compared to the newer studies, resulting in later presentation of GTD and higher rates of GTD-related complications such as hyperthyroidism. The higher rates of hyperthyroidism in this study may also reflect differences in healthcare systems and resources, as well as socioeconomic disparities.

Two studies examined the frequency of GTD-induced hyperthyroidism in a Middle-Eastern population. An Iraqi study found rates of biochemical hyperthyroidism to be as high as 25% (10/40) [14]. An Iranian study found rates of clinical hyperthyroidism to be similar to that shown in the UK and US study. Of 230 patients with a pathologically confirmed CM or PM, 10 were diagnosed with clinical hyperthyroidism (4.3%), all of whom had a complete mole [15].

#### Temporal trends of hyperthyroidism in GTD

There is some evidence that the clinical patterns and presentations of GTD have changed with the introduction of early pregnancy screening. We specifically explored temporal trends of hyperthyroidism in GTD.

A study by Hou et al. [6] compared 113 cases of hydatidiform mole during 1989–2006 with historical data from 1948 to 1975. They found significantly lower rates of GTD-related complications in the ‘modern’ cohort compared to the historical cohort, most likely due to earlier detection of GTD from routine use of first trimester ultrasonography and serum HCG testing [1, 6].

A Brazilian study [16] analysed medical records of women diagnosed with complete hydatidiform mole from 1988 to 2012. They assessed the prevalence of biochemical hyperthyroidism and trends over time. In contrast to the previous study by Hou et al., there was a significantly upward trend in the frequency of hyperthyroidism in women with GTD (0.69% in 1988–1992, 0.68% in 1998–2002 and 3.86% in 2008–2012). Notably, the latest rates (2008–2012) of biochemical hyperthyroidism at this centre was still lower than those described in the other studies. The significant increase in rates over time most likely reflects changes in management protocol for GTD. Prior to 2010, the centre only assessed patients for hyperthyroidism when patients presented with overt clinical hyperthyroidism, or when the uterus was markedly enlarged (> 16 cm). After 2010, however, routine screening for hyperthyroidism was conducted in all patients [16]. This example demonstrates that hyperthyroidism in GTD can be missed in the absence of routine biochemical screening.

#### Hyperthyroidism in complete mole versus partial mole

A Turkish cross-sectional study [17] assessed thyroid function in women with GTD, and compared results in PM with CM. Women with a pathologically confirmed diagnosis of CM had lower TSH (mean 0.28 vs 0.91; *p* = 0.011), higher free T4 (mean 2.15 vs 1.64; *p* = 0.042), and higher total T4 (mean 17.04 vs 2.04; *p* = 0.028) compared with those with pathologically confirmed PM. Of note, patients with complete mole were older and had more pregnancies than women with partial mole. This study only focused on biochemical hyperthyroidism and did not assess the women’s clinical status. The finding of higher rates of biochemical hyperthyroidism in CM corroborates with the results of the 2015 US study [12] described earlier.

#### Ethnicity and the risk of hyperthyroidism in GTD

We also investigated whether ethnicity was a risk factor for the development of hyperthyroidism in GTD. Only one 2016 retrospective study explored the effect of ethnicity on clinical features of complete mole. In all, 167 patients were studied, of which 96 (57%) were White ,22(15%) Asian, 22 (13%) Hispanic and 24 (14%) Black. No statistically significant association between race/ethnicity and frequency of hyperthyroidism was found [18].

### Pathophysiology of hyperthyroidism in GTD

The pathophysiological mechanisms underpinning hyperthyroidism in GTD have been explored by several studies. The hyperthyroid state in GTD cannot be explained by the effects of thyroid stimulating hormone (TSH) or thyroid stimulating antibodies (as in Graves’ disease) alone. In addition, removal of the hydatidiform mole or trophoblastic tumour results in the rapid resolution of hyperthyroidism. These findings point to the trophoblastic tissue as the main source of the thyroid stimulating agent [19].

hCG has thyrotropic activities and plays a central role in mediating hyperthyroidism in GTD [20–22]. This is due to “specificity spillover”, where one hormone interacts with the receptor of a different hormone, causing effects that are determined by the type of receptor activated [19]. Several conditions allow this “spillover” to occur. Firstly, hCG and TSH bear structural resemblance. hCG is made up of a heterodimer consisting of two subunits (alpha and beta) joined noncovalently. The alpha subunit of hCG is virtually identical to TSH, while its beta subunit is similar but sufficiently unique to give it its biological properties [23]. The similarities in biological structures between hCG and TSH allow hCG to exert its effects on the TSH receptor on thyroid membranes. Furthermore, the chances of a clinically significant “spillover effect” is increased during pathological states where there is an excess of the hormone, such as in GTD. In normal pregnancies, the affinity of hCG for the TSH receptor and thyrotropic potency of hCG is thought to be sufficiently low for the “spillover effects” to be negligible [19]. In support of this hypothesis, serum levels of hCG closely correlate with levels of thyroid hormone [24]. Importantly, this also suggests that serum hCG levels may predict the severity of clinical hyperthyroidism [11].

Several studies have also demonstrated that hCG secreted by hydatidiform moles and GTN have different biological properties from the hCG in women with normal pregnancies. Yoshimura et al. (1994) [25] found that the hCG isoform produced by hydatidiform moles had significantly greater thyrotropic activity compared to hCG preparations from normal pregnancy.

Kato and colleagues (2004) [26] focused on women who had hCG serum level lower than 100,000 IU/l and compared the frequency of hyperthyroidism between women with normal pregnancy, hydatidiform mole and choriocarcinoma. The authors found that patients with choriocarcinoma had a significantly higher incidence of hyperthyroidism (4/7; 57%) compared to patients with a normal pregnancy (5/28; 17.9%), which confirmed that hCG produced by choriocarcinomas may have altered biological properties. Of note, however, is that patients in the choriocarcinoma group had significantly higher levels of free beta-hCG compared to the normal pregnancy group. While the thyrotropic effect of free beta-hCG in the setting of choriocarcinoma is unclear, this may represent a potential confounder.

These findings corroborate the hypothesis that excessive secretion of a variant hCG molecule may underpin the pathogenesis of hyperthyroidism in GTD.

### Outcomes and complications of hyperthyroidism in GTD

Only case-reports and case-series have documented the outcomes and complications of hyperthyroidism in GTD.

The development of thyroid storm has been reported in cases of complete mole [27] and partial mole [28]. Thyroid storm refers to extreme clinical manifestations of thyrotoxicosis which can lead to organ decompensation. As objective scoring systems are not routinely available, it requires high level of suspicion and clinical acumen [29]. Of diagnostic importance is the fact that patients with hyperthyroidism in GTD usually do not exhibit classic features associated with Graves’ disease, such as ophthalmoplegia and pretibial myxoedema. This is likely due to the shorter duration of trophoblast-induced hyperthyroidism [27]. While some patients develop or present with clinical manifestations of thyroid storm during admission, there are also cases where thyroid storm developed after surgical evacuation of the molar pregnancy [30], likely due to the combination of high hCG levels, stress from the surgical procedure and hypovolemic state from blood loss. This emphasises the importance of pre-operative evaluation for hyperthyroidism [30] and careful anaesthetic considerations [31].

Treatment of hyperthyroidism in GTD is similar to that of hyperthyroidism due to primary thyroid pathologies. The majority of patients with clinical hyperthyroidism will respond to anti-thyroid medications and supportive care including beta blockers [11]. Furthermore, thyrotoxicosis resolves rapidly with mole evacuation, which is the definitive treatment modality. After surgery, virtually all patients become euthyroid, no longer have clinical manifestations of hyperthyroidism and do not require further medications in the weeks after discharge.

There have been a few reported cases of severe hyperthyroidism in GTD refractory to medical treatment strategies, requiring the use of therapeutic plasmapheresis to rapidly prepare the patients for surgery [32–34]. In one case of hydatidiform mole [33], thyroid hormone levels remained high despite treatment with propylthiouracil and propranolol. Despite the addition of cholestyramine and inorganic iodine, hyperthyroidism persisted on day 14. In another reported case of a partial mole [34], levels of thyroid hormone remained high despite 10 days of treatment with propylthiouracil and propranolol, and the addition of inorganic iodine and dexamethasone. In addition, the second patient experienced increasing amounts of vaginal bleeding. With urgent surgery indicated for these two cases, rapid hormonal and haemodynamic control was critical as preoperative thyrotoxicosis is associated with surgical morbidity and mortality [35]. The administration of plasmaphoresis in both cases resulted in a rapid decrease in thyroid hormone levels, due to removal of excess thyroid hormone bound to serum proteins. Therapeutic plasmaphoresis may therefore be considered in patients who either fail to respond to medical therapy, or who develop complications from medications. Although these two patients did not develop side effects from the procedure, plasmaphoresis may be associated with complications including pruritus, urticaria, hypotension, coagulopathy and anaphylaxis [36].

While the outcomes of hyperthyroidism in GTD are generally good, a number of case reports have described severe complications, including thyroid storm-induced multi-organ failure [37], ARDS, pulmonary oedema [38], pulmonary hypertension [39] and acute renal failure [40].

### Summary of findings

Hyperthyroidism is a potentially life-threatening complication of GTD. The pathophysiology of trophoblast-induced hyperthyroidism has been well-elucidated, and it can be explained by the structural homology of hCG and TSH, excessive hCG levels secreted by trophoblast in GTD and the increased thyrotropic activity of hCG in GTD. Biochemical hyperthyroidism is more likely to be observed in CM compared to PM, which relates to the greater amount of hCG produced in CM.

The incidence and prevalence of hyperthyroidism is likely to be significantly affected by implementation and uptake of early antenatal screening. The increasing use of first trimester ultrasonography and serum hCG testing combined with early management has led to fewer women presenting with GTD late in gestation, thus lowering the frequency of characteristic GTD symptoms such as hyperthyroidism. The rates of hyperthyroidism in GTD may also serve as a marker for socioeconomic disparities between healthcare systems and healthcare resources. There is insufficient evidence to assess ethnicity as a risk factor for hyperthyroidism in GTD, although future studies should confirm this.

It is important to detect and manage hyperthyroidism as it can lead to significant morbidity and mortality. Most cases of hyperthyroidism in GTD can be effectively controlled by anti-thyroid medications, and plasmaphoresis may represent an alternative therapeutic option in patient’s refractory to medical treatment or patients requiring urgent surgery. Surgical evacuation of the uterus remains the only definitive treatment option for hyperthyroidism in GTD. Most patients will be euthyroid and not require further antithyroid treatment after surgery. Prior to surgery, however, a comprehensive anaesthetic work-up for hyperthyroidism is critical. Hyperthyroidism is an important perioperative consideration in patients with GTD, and haemodynamic status and thyroid function should be optimised prior to surgery, in order to prevent complications. Careful planning of perioperative anaesthetic management is also vital [41].

The paucity of observational studies on this topic meant that a quantitative exploration (meta-analysis) of our outcomes could not be performed. There was also considerable heterogeneity between the studies, particularly in the case definition for GTD, diagnostic criteria for hyperthyroidism and diversity of patient populations (e.g. timing of presentation, pre-existing comorbidities and type of GTD). The retrospective nature of several observational studies also makes them susceptible to information bias, as study variables and outcomes could be misclassified. In addition, it is difficult to verify the accuracy and completeness of recorded information. For example, patients with missing information relating to biochemical markers of thyroid function may have been excluded, leading to selection bias.

## Conclusion

In this review, we have shown that hyperthyroidism is a rare but important clinical entity in GTD. Though highly treatable, it may result in substantial morbidity and mortality. Larger observational studies are needed to deepen our understanding of this condition, improve early detection and reduce its associated complications.

## Supplementary Information

**Additional file 1.** Complete search strategy for all databases. Description: This file contains the search strategy that we used for Medline, EMBASE and Cochrane Library.

## Data Availability

Data sharing is not applicable to this article as no datasets were generated or analysed during the current study.

## Abbreviations

GTD: Gestational trophoblastic disease
GTN: Gestational trophoblastic neoplasia
PSTT: Placental site trophoblastic tumour
ETT: Epithelioid trophoblastic tumour
TSH: Thyroid stimulating hormone
hCG: human chorionic gonadotropin
ARDS: Acute respiratory distress syndrome
