# Supplementary material for: Hyperthyroidism in gestational trophoblastic disease – a literature review
Source: Thyroid Res. 2021 Jan 14;14:1. doi: 10.1186/s13044-021-00092-3 (PMC7807451; doi:10.1186/s13044-021-00092-3)
Supplement: Supplementary file 1 — Additional file 1. Complete search strategy for all databases. Description: This file contains the search strategy that we used for Medline, EMBASE and Cochrane Library. [file 13044_2021_92_MOESM1_ESM.docx]

Additional File 1

| **Medline (from database inception to 13^th^ October 2019)**   1. gestational trophoblastic.mp. 2. molar pregnancy.mp. 3. hydatidiform mole.mp. 4. complete mole.mp. 5. partial mole.mp. 6. choriocarcinoma.mp. 7. placental site trophoblastic.mp. 8. Epithelioid trophoblastic.mp. 9. gestational trophoblastic disease/ 10. trophoblastic neoplasms/ 11. 1 OR 2 OR 3 OR 4 OR 5 OR 6 OR 7 OR 8 OR 9 OR 10 12. hyperthyroidism.mp. 13. thyrotoxicosis.mp. 14. thyroid storm.mp. 15. hyperthyroidism/ 16. 12 OR 13 OR 14 OR 15 17. 11 AND 16   **EMBASE (from database inception to 13^th^ October 2019)**   1. gestational trophoblastic.mp. 2. molar pregnancy.mp. 3. hydatidiform mole.mp. 4. complete mole.mp. 5. partial mole.mp. 6. choriocarcinoma.mp. 7. placental site trophoblastic.mp. 8. Epithelioid trophoblastic.mp. 9. exp hydatidiform mole/ 10. exp trophoblastic tumor/ 11. 1 or 2 or 3 or 4 or 5 or 6 or 7 or 8 or 9 or 10 12. hyperthyroidism/ or subclinical hyperthyroidism/ or thyroid crisis/ or thyrotoxicosis/ 13. hyperthyroidism.mp. 14. thyrotoxicosis.mp. 15. thyroid storm.mp. 16. 12 or 13 or 14 or 15 17. 11 and 16   **Cochrane library (from database inception to 13^th^ October 2019)**   1. gestational trophoblastic disease 2. [gestational trophoblastic disease] MeSH 3. molar pregnancy 4. hydatidiform mole 5. complete mole 6. partial mole 7. choriocarcinoma 8. placental site trophoblastic 9. epithelioid trophoblastic 10. #1 OR #2 OR #3 OR #4 OR #5 OR #6 OR #7 OR #8 OR #9 11. [hyperthyroidism] MeSH 12. Hyperthyroidism 13. Thyrotoxicosis 14. thyroid storm 15. #11 or #12 or #13 or #14 16. #10 AND #15 |
| --- |
